# Investigation on Wear Behavior of Cryogenically Treated Ti-6Al-4V Titanium Alloy under Dry and Wet Conditions

**DOI:** 10.3390/ma12182850

**Published:** 2019-09-04

**Authors:** Yonggang Li, Xingfu Wang, Shengqiang Yang, Lifeng Hou, Yinghui Wei, Zhongjie Zhang, Xiaoni Yang

**Affiliations:** 1 College of Mechanical and Vehicle Engineering, Taiyuan University of Technology, Taiyuan 030024, China; 2 Taiyuan Heavy Machinery Group Co., Ltd., Taiyuan 030024, China; 3 College of Materials Science and Engineering, Taiyuan University of Science and Technology, Taiyuan 030024, China; 4 College of Water Resources Science and Engineering, Taiyuan University of Technology, Taiyuan 030024, China

**Keywords:** Ti-6Al-4V alloy, cryogenic treatment, wear behavior

## Abstract

Titanium alloys are widely used in many fields because of their excellent comprehensive properties. However, its poor friction and wear properties limit its many potential applications. In general, the surface roughness of important parts manufactured by titanium alloy should meet certain requirements. As a low-cost and high-efficiency processing method, barrel finishing has been used for the surface finishing of titanium alloys. The main material removal mechanism of barrel finishing is micro-cutting/grinding, which is similar to the material wear mechanism under some conditions. In addition, titanium alloys are subjected to a low force in common surface finishing processes. Cryogenic treatment is a method that can improve the comprehensive properties of titanium alloys. Therefore, the friction and wear behavior of cryogenically treated Ti-6Al-4V titanium alloy (CT Ti alloy) and non-cryogenically treated Ti-6Al-4V titanium alloy (NT Ti alloy) at a low load and scratch speed was studied comparatively in this paper. The results show that the CT Ti alloy exhibits a lower friction coefficient and wear rate under both dry and wet wear conditions. Under wet conditions, the stabilized friction coefficient is lower than that under dry conditions. The stabilized friction coefficient of CT Ti alloy is 0.18 after reaching a stable wear stage under wet conditions. Under dry wear conditions, the NT Ti alloy mainly showed typical abrasive wear, heavy adhesion wear and oxidation wear characters. The wear mechanisms of CT Ti alloy are mainly abrasive wear, slight adhesion wear and oxidation wear. Under wet wear conditions, the wear mechanism of NT Ti alloy is abrasive wear and slight adhesion wear. After cryogenic treatment, the mechanism for CT Ti alloy is slight abrasive wear.

## 1. Introduction

Titanium alloys are favored in the production of aerospace parts due to their excellent comprehensive properties [1]. The demand and application of these alloys has been increased in other fields such as transportation, the petrochemical industry, biomedicine and daily life, etc. [2]. However, it also exposes many shortcomings and problems in the process of practical application. The shortcomings, like relatively low hardness, easy adhesion, poor thermal conductivity, low wear resistance, poor fatigue resistance and other defects, will greatly limit its popularization and application [3,4]. Some literatures show that the failure of titanium alloy parts is usually related to surface wear and corrosion [5]. Therefore, a comprehensive and in-depth study on the wear problem of titanium alloys is of great importance and significance. In view of the wear problem of titanium alloys, scholars have mainly focused on two aspects of research works. On the one hand, it is to improve the surface wear resistance of titanium alloy. On the other hand, it is to study the wear mechanism. In terms of wear resistance, researchers mainly apply the following means to improve the wear resistance of titanium alloys, such as particle reinforcement [6,7], laser cladding [8,9,10], surface plastic deformation [11,12,13], electrodeposition [14], ion implantation [15,16,17], anodic oxidation [18], magnetron sputtering [19], electrolytic/thermal oxidation [20,21], etc. In the aspect of wear mechanism, the main wear forms of titanium alloys were studied, including fretting wear, erosion wear, fatigue wear, adhesion wear, abrasive wear and corrosion wear. Sometimes the actual situation may be that some of these wear forms act simultaneously. Generally, with the change of actual working conditions, the influence degree of these wear forms also changes.

In recent years, the friction and wear properties of titanium alloys under dry, wet, corrosion, high temperature and lubrication conditions have attracted extensive attention of researchers. Straffelini et al. [22] investigated the dry friction and wear behavior of Ti-6Al-4V titanium alloy using the high speed steel as pairing material at room temperature. The results show that the wear mechanism transformation of the alloy occurs in the scratch speed range of 0.4–1.0 m/s, and the wear rate decreases with the increase of scratch speed. Liu et al. [23] studied the dry friction and wear properties of Ti-6Al-4V titanium alloy under low temperature and vacuum conditions. The results show that low temperature causes micro-cracks at wear marks at a low scratch speed. At a high scratch speed, micro-cracks do not occur. Cvijovic-Alagic et al. [24] studied the friction and wear properties of Ti-6Al-4V titanium alloy in simulated body fluids. It was found that the wear of Ti-6Al-4V alloy increased with the increase of scratch speed. Feng et al. [25] studied the effect of quenching treatment on the wear behavior of Ti-6Al-4V titanium alloy. The results show that there is no obvious change in the microstructure and hardness of Ti-6Al-4V titanium alloy after quenching at 750 °C. When quenched above the temperature of the β phase transition, the hardness of the alloy increases significantly from 400 HV to 800 HV. However, the wear resistances of the quenched alloys with higher hardness decrease due to the change of wear mechanism from plastic deformation to brittle fracture. The effect of microstructure on wear behavior of Ti-6Al-4V titanium alloy was studied by Hadke et al. [26]. The results show that the hardness of the alloy increases and wear resistance decreases after quenching. The ability to resist abrasive wear is mainly affected by microstructure rather than hardness. The hardness of the original alloy is low, and the abrasive wear is mainly caused by micro-ploughing. The material removal mechanism of quenched samples is mainly caused by micro-cutting. Li et al. [27] studied the wear behavior of Ti-6Al-4V alloy in the sliding speed range of 0.5–4 m/s. The results showed that the wear rate changed in three stages: First slowly decreasing (0.5–1.5 m/s), then rapidly increasing until the sliding speed reached 2.68 m/s, and then decreasing again (2.68–4 m/s). When the sliding speed was 4 m/s, the wear rate was the lowest. The wear mechanisms in the above three stages are mainly delamination wear and oxidation wear at a low sliding speed, delamination wear at a medium speed and oxidation wear at a high speed. The adhesion wear, delamination wear and abrasive wear are the main wear types of Ti-6Al-4V alloy at 25–200 °C [28]. In the high temperature range of 400–500 °C, oxidation wear is the main wear mechanism [28]. The experiments carried out by Cui et al. [29] show that the wear rate of Ti-6Al-4V alloy increases linearly with the load at 20 °C. When the temperature is 400 °C and the load is 50–100 N, the wear rate decreases gradually with the increase of the load. When the load is in the range of 100–200 N, the wear rate increases with the increasing of the load. If the load is greater than 200 N, the wear rate of the alloy increases rapidly. Frictional oxide plays a very good role in reducing wear during the wear process at 400 °C. However, the investigation carried out by Wang et al. [30] showed that the dispersed frictional oxide had little antifriction effect when the load was in the range of 50–250 N and the temperature was in the range of 25–200 °C. For Ti-6.5Al-3.5Mo-1.5Zr-0.3Si alloy, frictional oxide takes a positive effect on reducing friction when the temperature is between 500 °C and 600 °C. It is indicated that the chemical composition has a significant effect on the high temperature wear behavior of titanium alloys. Komotori et al. [31] found that the wear mechanism of Ti-6Al-4V alloy changed from abrasive wear to adhesive wear with the increase of friction speed. High friction speed hinders the formation of passive film at the wear mark and leads to pitting formation. Friction speed is an important factor affecting the wear failure process. Sahoo et al. [32] investigated the erosion wear resistance of Ti-6Al-4V titanium alloy. The impact velocity is the most important factor affecting the erosion wear of Ti-6Al-4V titanium alloy, and the change of microstructure has little effect on wear. This research team also studied the effect of friction factors on wear rate. When the load was between 50 N and 150 N, the scratch speed was between 0.3 m/s and 0.9 m/s and the test time was between 30 min and 60 min, and the effect of these friction factors on wear weight loss showed the following rules: Pressure (load) > scratch speed > test time > microstructure. The oxidation wear occurs at a low scratch speed, while the delamination wear occurs at a high scratch speed [33]. In the aspect of friction reduction of titanium alloys, the friction and wear properties of titanium alloys can be improved by choosing suitable kinds of solid lubricants, particle sizes and concentrations [34,35].

Among many surface modification technologies, cryogenic treatment can improve the strength, toughness, wear resistance and corrosion resistance of titanium alloys comprehensively [36,37]. Moreover, the stability of mechanical processing can be improved by cryogenic treatment [38,39]. However, there are few studies on the friction and wear properties of cryogenically treated titanium alloys. Especially the investigations carried out under the conditions of low load and scratch speed. In fact, the wear conditions mentioned above are similar to those of barrel finishing. The mechanism of material removal of wear is also similar to that of barrel finishing and is micro-cutting/grinding. In various forms of the barrel finishing process, the force between the workpiece and abrasive particle is about 5 N, and the relative velocity of the two parts is about 0.05 m/s [40]. Therefore, the study of the friction and wear process and mechanism of titanium alloy under low load and scratch speed conditions will be helpful to improve the barrel finishing process of titanium alloy. It will provide a basis for realizing low cost and high efficiency surface processing for titanium alloy.

As a result, Ti-6Al-4V titanium alloy was used as the research material, and then it was placed in a liquid nitrogen cryogenic tank for cryogenic treatment. The friction and wear experiments of CT Ti alloy and NT Ti alloy were carried out under dry and wet wear conditions. The hardness, microstructure, wear rate, friction coefficient, surface morphology and wear mechanism of the samples before and after cryogenic treatment were systematically analyzed by means of a hardness tester, scanning electron microscopy (SEM), energy dispersive spectrometer (EDS) and 3D profiler.

## 2. Materials and Methods

### 2.1. Material and Cryogenic Treatment

Ti-6Al-4V titanium alloy was chosen as the research material in the experiment, and its composition is shown in Table 1. It was machined into a sample with a size of 20 mm × 20 mm × 10 mm. Before the experiment, all Ti-6Al-4V titanium alloy samples were manually ground with sandpaper, then cleaned with ethanol and dried. The cryogenic treatment of titanium alloy was carried out in a CDW-196 liquid nitrogen cryogenic tank. Titanium alloy specimens were subjected to cryogenic treatment for 24 h, and then recovered to room temperature for subsequent testing and analysis.

### 2.2. Wearing Test

The reciprocating friction and wear experiments of Ti-6Al-4V titanium alloy under dry/wet conditions were carried out with a CFT-I multifunctional friction and wear tester (Lanzhou Zhongke Kaihua Technology Development Co., Ltd., Lanzhou, China) at room temperature. ZrO_2_ ceramic balls with a diameter of 3 mm were selected for the other friction pair material. The reciprocating length was 5 mm. Five locations on the wear mark were selected for measurement, and the average value was taken as the wear amount of the wear mark. The wear rate was calculated as follows [41]:

*Wr* = Δ*V/L* (1)

where *Wr* is the wear rate of the test material, mm^3^/m; Δ*V* is the wear volume, mm^3^; and *L* is the wear distance, m.

The main components of liquid media used in wet friction and wear experiments are sodium alcohol ether sulphate (AES), oleic acid, coconut oil fatty acid diethanolamin and primary alcohol ethoxylate (AEO). The liquid media formula shows as follows: Deionized water 1000 mL, each liquid media 50 mL. The formula is always used for barrel finishing of titanium alloy.

### 2.3. Surface Analysis

The microstructure of titanium alloy was analyzed by SEM (TESCAN, Brno, Czech Republic). Before the experiment, titanium alloy samples were ground with sandpaper, then polished and etched. The chemical etchant formula used in the experiment is a mixture of 90 mL deionized water, 2 mL hydrofluoric acid (HF) and 10 mL concentrated nitric acid (HNO_3_). The surface hardness of the workpiece before and after cryogenic treatment was measured by an HMV-G21ST hardness tester (Shimadzu (Asia Pacific) Pte. Ltd., Kyoto, Japan). During the experiment, the load was 10 N and the testing time was 15 s. Seven points were taken from each sample for measurement, and then the average value was taken as the final result. SEM was used to analyze the surface morphology of wear samples, and EDS was used to study the composition of wear marks and wear debris. The main working parameters of the SEM were an accelerating voltage of 30 kV and a working distance of 15 mm. The phase composition of the Ti-6Al-4V titanium alloy was analyzed via TD3600 X-ray diffraction (XRD, Dandong Tongda Science and Technology Co., Ltd., Dandong, China) equipped with a Cu target. The generator was set for 40 kV and 30 mA. Data was collected between 2θ = 30° and 80° at 0.02° intervals. The three-dimensional micro-morphology of wear marks on the surface of titanium alloy was observed by an SM-1000 laser copolymerization three-dimensional profilometer (Sixian Photoelectric technology (Shanghai) Co.,Ltd., Shanghai, China). 

## 3. Results and Discussion

### 3.1. Effect of Cryogenic Treatment on the Microstructure of Ti-6Al-4V Alloy

**Figure 1:**
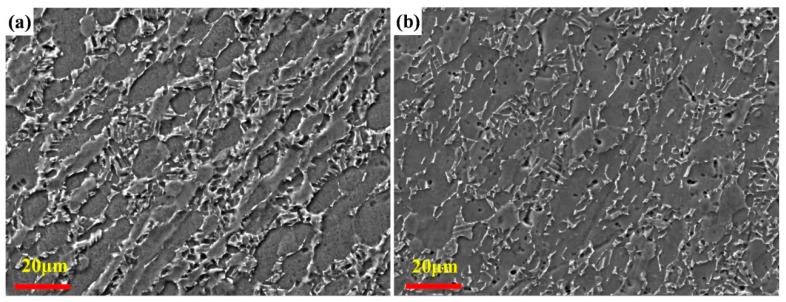
The microstructure of Ti-6Al-4V alloy: (**a**) as-received alloy (NT Ti alloy) and (**b**) after cryogenic treatment for 24 h (CT Ti alloy).

**Figure 2:**
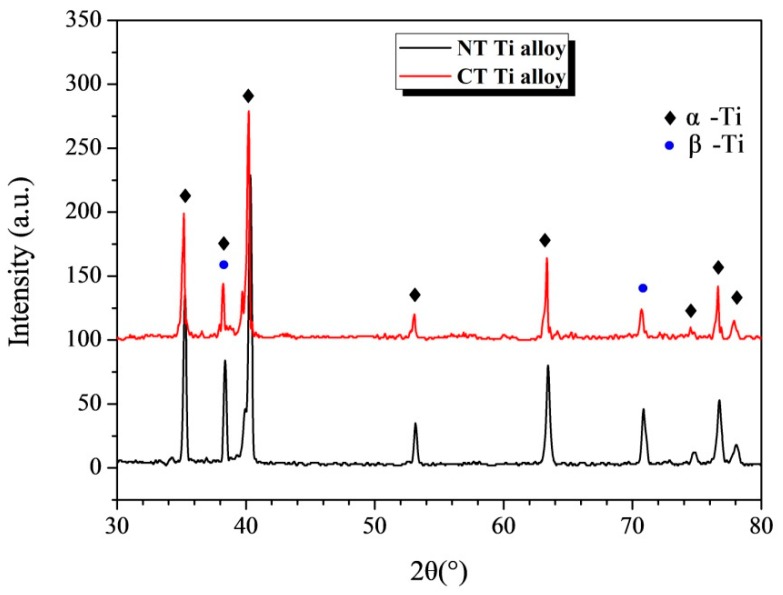
The X-ray diffraction (XRD) patterns of Ti-6Al-4V alloy with and without cryogenic treatment.

Figure 1a shows the microstructures of the NT Ti alloy. It consists of two phases, the coarse α phase is dark gray, and fine β phase is bright white. Figure 1b shows the microstructures of the alloy after cryogenic treatment for 24 h. Compared with the microstructures of the as-received material, the number and size of the β phase of CT Ti alloy decrease slightly. This is consistent with the XRD results in Figure 2. Further analysis of XRD data shows that the content of the β phase in the NT Ti alloy samples is 19.5%, and that after cryogenic treatment for 24 h, the content of the β phase in the CT Ti alloy samples is 15%. Cryogenic treatment results in the decrease of β phase content in the alloys. This is consistent with the microstructure shown in Figure 1. The ambient temperature of the sample during cryogenic treatment is very low, which makes the volume of the material shrink. The investigation carried out by Li et al. shows that the pressure of Ti-6Al-4V titanium alloy specimens bear during cryogenic treatment (−196 °C) is about 205 MPa/cm^2^ [42]. Under the action of enormous external force, part of the β phase is transformed into a fine secondary α phase, which reduces the content of the β phase to a certain extent [43]. At the same time, after cryogenic treatment, some defects and internal stress of the titanium alloy will be improved, which greatly enhances the stability of the material microstructure, thereby improving its mechanical properties [44].

### 3.2. Effect of Cryogenic Treatment on the Microhardness of Ti-6Al-4V Alloy

**Figure 3:**
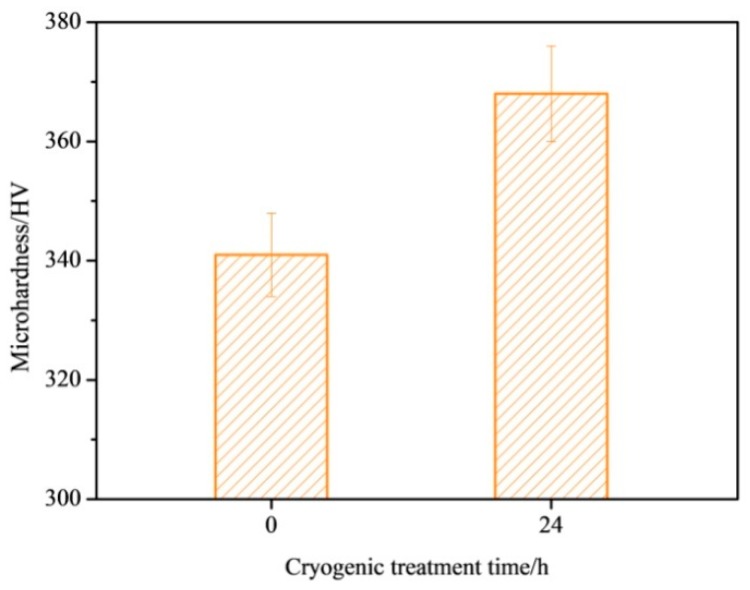
The microhardness of Ti-6Al-4V alloy with and without cryogenic treatment.

Figure 3 is a histogram of the microhardness test result of Ti-6Al-4V titanium alloy at different cryogenic treatment times. The hardness of the NT Ti alloy sample is 341 HV. After 24 h cryogenic treatment, the microhardness of the titanium alloy sample increased to 368 HV, which was 7.9% higher than that of the untreated samples. In the two phases of Ti-6Al-4V alloy, the microhardness of the α phase is higher than that of the β phase. Moreover, the compressive stress during cooling leads to the increase of dislocation density and a more stable lattice structure, which results in the increase of microhardness of the specimens after cryogenic treatment [45,46]. 

### 3.3. Effect of Cryogenic Treatment on the Wear Behavior of Ti-6Al-4V Alloy

#### 3.3.1. Effect of Cryogenic Treatment on the Friction Coefficient of Ti-6Al-4V Alloy

**Figure 4:**
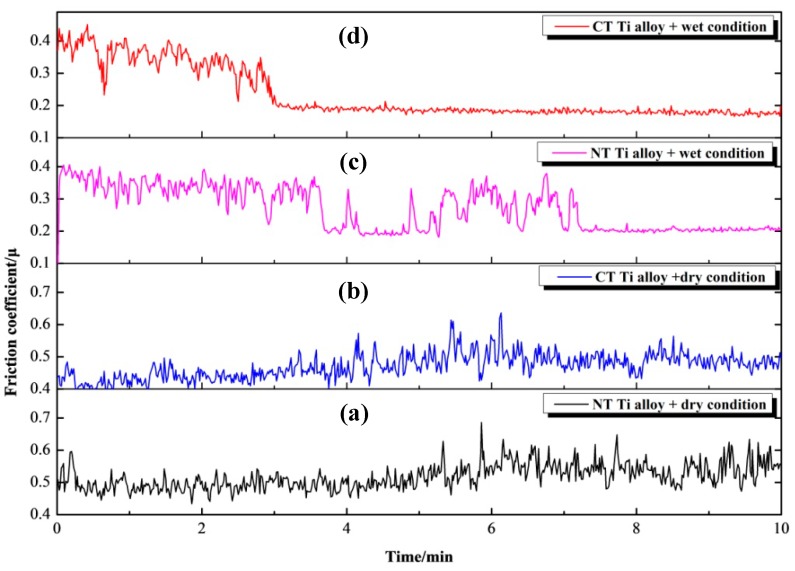
The friction coefficients of Ti-6Al-4V alloy with and without cryogenic treatment.

Figure 4 shows the friction coefficient curves of NT Ti alloy and CT Ti alloy samples under dry and wet conditions with a load of 5 N and a sliding speed of 5 cm/s. From Figure 4a, it was observed that the friction coefficient of the NT Ti alloy samples increased slowly with the prolongation of loading time within 0–6 min. When the loading time was extended to 6 min, the coefficient value was about 0.55. Then there was a sudden change, and the value increased to a certain extent. The friction coefficient gradually stabilized 7 min later. The friction coefficient fluctuated within 0–10 min. The range of change is a little larger than that gained from the wet condition. The overall range of change was between 0.4–0.6. After 24 h of cryogenic treatment, it can be seen from Figure 4b that the trend of friction coefficient change of CT Ti alloy specimens is similar to that of NT Ti alloy specimens in 3 min. The friction coefficient of CT Ti alloy increases in 3–5 min. Then the coefficient decreases slightly and enters a stable stage in 5–10 min. It is indicated that the wear process also gradually enters a stable stage. The overall variation range of the friction coefficient was between 0.4 and 0.5. The reasons for the change of the friction coefficient of titanium alloy samples are as follows: In the initial stage of friction, the adhesive wear is prone to occur on the surface of titanium alloy because the hardness of titanium alloy samples is less than that of ZrO_2_ balls. Moreover, the contact area of the two friction pair materials is small, resulting in large contact stress. The friction coefficient will increase to a certain extent after the adhesive wear occurs, and then tend to be stable [47].

Figure 4c,d are the time-varying curves of the friction coefficients of Ti-6Al-4V titanium alloy before and after cryogenic treatment under wet conditions with a load of 5 N and a sliding speed of 5 cm/s. The results show that the friction coefficients of NT Ti alloy and CT Ti alloy samples are larger at the initial stage of wear. With the increase in loading time, the friction coefficients decreased obviously in both of the two groups of samples. Finally, the friction coefficients of NT Ti alloy samples tend to be 0.2, while the friction coefficients of CT Ti alloy samples were stabilized at 0.18. There is little difference in friction coefficient between the two groups of samples at the stable friction stage. However, the time needed to reach steady wear is different for these two kinds of specimens. After cryogenic treatment for 24 h, the friction coefficient of the CT Ti alloy samples reached 0.18 after 3 min. By comparison, the friction coefficient of the NT Ti alloy specimens was stabilized at 0.2 after 7 min. At the same time, it can be seen that the friction coefficient of the NT Ti alloy samples suddenly decreases during the period of 4–5 min, and then there is an obvious fluctuation. Some literatures show that even under lubrication conditions, adhesion wear of titanium alloys can occur [48], which is related to the contact state of friction pair materials in the initial stage of friction. With the progress of the experiment, a certain amount of debris is produced on the surface of the titanium alloy matrix, which plays the role of lubrication, isolation and support together with the liquid medium. As a result, the friction coefficient decreases to a certain extent. Therefore, it can be inferred that the adhesive wear degree of NT Ti alloy samples is more serious than that of CT Ti alloy samples under wet conditions. With the increase in loading time, the friction coefficients of NT Ti alloy samples and CT Ti alloy ones are all close to about 0.2.

By comparing the variation in the friction coefficient after stabilization under dry and wet friction and wear conditions, it is found that the fluctuation range of the friction coefficient in dry conditions is larger than that in wet conditions. This is because the process from debris formation to debris leaving the friction system under dry conditions is relatively unstable compared with that under wet conditions. The variation range of the friction coefficient of CT Ti alloy is lower than that of NT Ti alloy, the coefficient value is smaller, and the friction and wear process is more stable. In this experiment, the friction and wear process of cryogenically treated samples is the most stable one under wet wear conditions.

#### 3.3.2. Effect of Cryogenic Treatment on the Wear Rate of Ti-6Al-4V Alloy 

**Figure 5:**
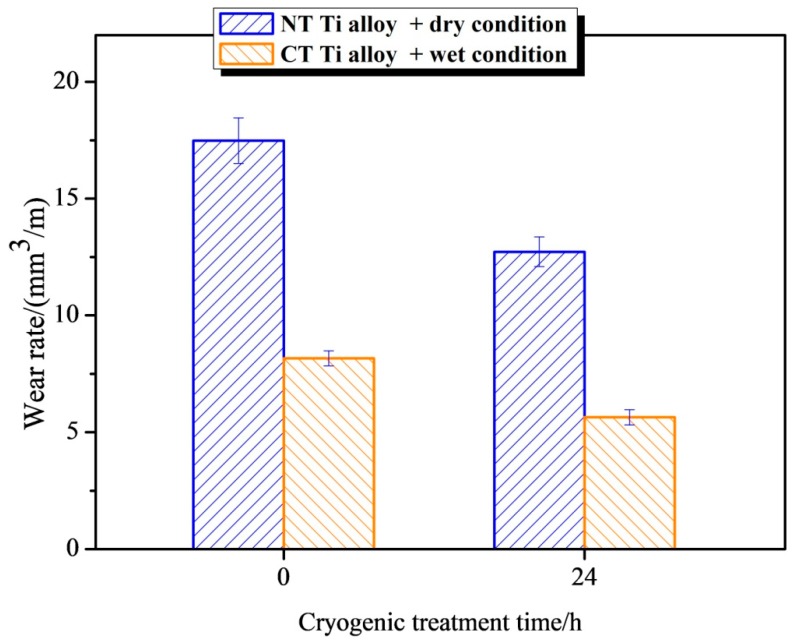
The wear rates of Ti-6Al-4V alloy with and without cryogenic treatment.

Figure 5 is a histogram of the wear rate of NT Ti alloy and CT Ti alloy under dry and wet wear conditions. The figure shows that, under dry wear conditions, the wear rate of CT Ti alloy samples is 31.5% lower than that of NT Ti alloy ones. After cryogenic treatment, the wear resistance of the titanium alloy under dry conditions can be improved. This is because, in general, higher hardness can give the material a better wear resistance [49,50].

It can be seen from the figure that, under wet conditions, the wear rate of CT Ti alloy samples is 30.9% lower than that of NT Ti alloy samples. The wear resistance is obviously improved by cryogenic treatment. By comparison, it is found that the wear rates of CT Ti alloy samples under both dry and wet wear conditions are significantly lower than those of NT Ti alloy. The wear rate of the samples with and without cryogenic treatment is smaller under wet conditions, which is 54.3% and 53.6% lower than that under dry conditions, respectively.

#### 3.3.3. Effect of Cryogenic Treatment on the Wear Morphologies and Mechanisms of Ti-6Al-4V Alloy

**Figure 6:**
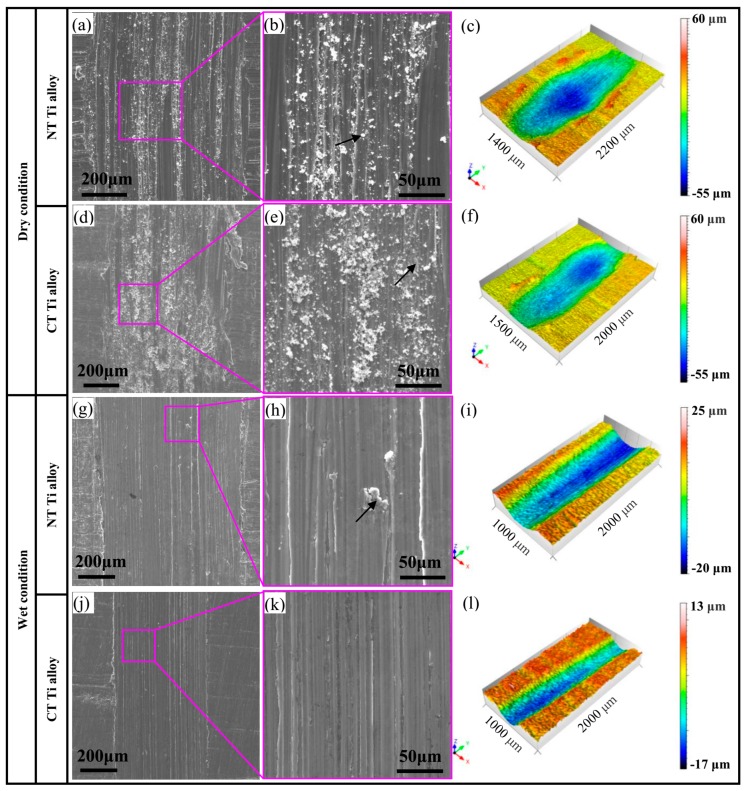
The wear morphologies of Ti-6Al-4V alloy before and after cryogenic treatment under dry/wet condition: (**a**–**c**) the SEM morphologies and the 3D morphology of NT Ti alloy under dry conditions; (**d**–**f**) the SEM morphologies and the 3D morphology of CT Ti alloy under dry conditions; (**g**–**i**) the SEM morphologies and the 3D morphology of NT Ti alloy under wet conditions; and (**j**–**l**) the SEM morphologies and the 3D morphology of CT Ti alloy under wet conditions.

Figure 6a–f are the worn surface morphologies of Ti-6Al-4V alloy samples with and without cryogenic treatment under dry friction and wear conditions with a load of 5 N and a sliding speed of 5 cm/s. Figure 6a,b show that the plough grooves on the surface of the NT Ti alloy samples are fine and dense, and a large number of white debris are attached to the surface of the wear marks. The layer-like protrusions in middle of the wear marks are formed due to the adhesion effect. Figure 6d,e reveal that the surface of the CT Ti alloy sample is relatively flat. The shallow plough grooves and slight adhesion marks distribute on the worn surface. Moreover, the width of the wear marks is narrower and shallower than that of NT Ti alloy. In addition, the wear characteristics of CT Ti alloy samples are similar to those in the literature [43].

Figure 6c,f are 3D profiles of wear marks of NT Ti alloy and CT Ti alloy samples under dry friction. It can be seen that the wear marks of the titanium alloy matrix after the wear test are basically long pits. There are clearly visible plastic deformation zones turning to both sides of the pit edges. After cryogenic treatment for 24 h, the depth of the wear scar profile of the titanium alloy is shallower than that of the non-cryogenic treated sample. The wear volume of the CT Ti alloy specimen is smaller, and the plastic deformation zones on both sides of the wear scar are not obvious. The results stated above indicate the wear rate of CT Ti alloy is lower than that of NT Ti alloy, which is basically consistent with the results shown in Figure 6.

Figure 6g–l are the worn surface morphologies of Ti-6Al-4V alloy samples under wet friction and wear conditions. There are obvious plough groove and slight adhesive wear on the surface of the NT Ti alloy sample shown in Figure 6g,h. The width of the wear mark is larger than that shown in Figure 6j. It can be seen from Figure 6j,k that the worn surface of the CT Ti alloy sample is relatively flat. Many shallow furrows distribute on the wear scar surface. There are no signs of adhesive wear. The width of the wear marks is narrower and the depth is shallower.

Figure 6i,l are the 3D morphologies of wear marks of Ti-6Al-4V titanium alloy samples with and without cryogenic treatment under wet conditions. It can be seen from Figure 6i that the wear scar morphology of the titanium alloy matrix after the wear test is basically the shape of the long pit under the condition of liquid lubrication. There are clearly visible plastic deformation zones turning to both sides of the pit edges. By comparison, the much slighter wear was observed on the surface of the CT Ti alloy. The main wear features are as follows: The depth and width of the wear marks are shallower, the wear volume is smaller and the plastic deformation zones on both sides of the wear marks are not obvious. 

Compared with the surface morphology of wear marks under dry and wet wear conditions, it can be found that the surface of wear marks under wet wear conditions is smoother. There are fewer furrows, slighter adhesion phenomenon, smaller wear volume, shallower and narrower wear marks and a more stable wear process. It is indicated that the wear resistance of the alloy is higher under wet condition. The CT Ti alloy samples have better wear resistance than NT Ti alloy ones under dry/wet conditions. This is because cryogenic treatment can improve the hardness and processing stability of Ti-6Al-4V titanium alloy, and these factors will help to improve its wear resistance.

**Figure 7:**
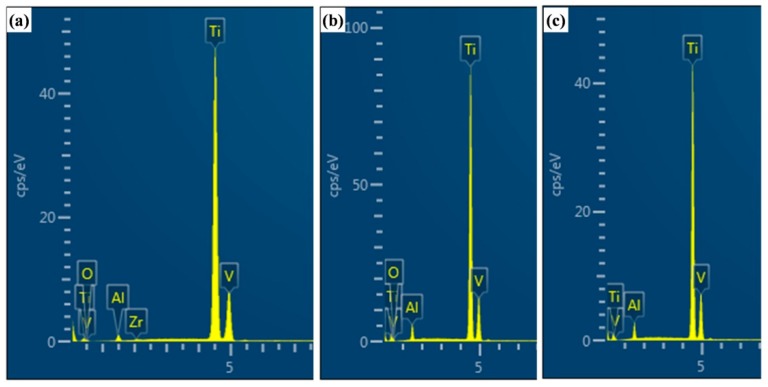
EDS results of a typical area on the worn surface in Figure 6.

The results of the chemical composition analysis for typical wear areas are shown in Figure 7. Figure 7a is the EDS result of white debris in Figure 6b. The results show that there are Zr and O elements in the debris. The transfer of the Zr element to debris indicates the occurrence of adhesive wear. Figure 7b is an analysis of the pale area in Figure 6e that will be peeled off from the matrix. This area contains not only the element of the matrix, but also the O element. EDS analysis was carried out on the middle position of the dark gray area to be peeled off from the matrix in Figure 6h. The results showed that the main composition was the elements in the matrix (Figure 7c). According to the comprehensive analysis of Figure 6 and Figure 7, the wear mechanism of NT Ti alloy samples are the combination of typical abrasive wear, heavy adhesion wear and slight oxidation wear under dry wear condition. The wear mechanism for CT Ti alloy samples are abrasive wear, slight adhesion wear and oxidation wear. The results show that the wear of CT Ti alloy is slighter than that of NT Ti alloy, and the wear process is more stable. The wear resistance of Ti-6Al-4V titanium alloy is improved by cryogenic treatment. Under wet wear conditions, the wear mechanism of samples without cryogenic treatment is abrasive wear and slight adhesion wear. After cryogenic treatment, the abrasive wear is the main mechanism for CT Ti alloy.

When the hardness of the material itself is high, the abrasive wear is prone to occur, and when the hardness is low, adhesive wear will be appearing [51]. The higher the contact stress, the easier the adhesive wear will occur [52]. If the contact stress is high and exceeds a 1/3 of the hardness of the material, adhesive wear will easily occur. In addition, cryogenic treatment results in more dislocations in titanium alloy samples [42,45], which improves the resistance to micro-plastic deformation. These factors together lead to slighter adhesion wear of CT Ti alloy samples. If the scratching speed is low, the oxidation wear of the titanium alloy will also occur at a low temperature [33]. Therefore, under dry friction and wear conditions, abrasive wear, adhesion wear and slight oxidation wear will occur on titanium alloys. In the initial stage of wear, due to the small contact area and large contact stress between the friction pair, under the action of shear stress in the friction process, the materials on the surface of the titanium alloy and the surface of the ZrO_2_ ball transfer mutually. The material transfer process is mainly from the surface of the titanium alloy sample to that of the ZrO_2_ ball. At this time, micro-convex bodies will be formed on the ZrO_2_ ball, and these micro-convex bodies will have a serious ploughing effect on the titanium alloy samples. Therefore, the friction coefficient increases slowly in the initial stage of friction. As the friction process proceeds, the micro-convex body will harden and fall off from the ZrO_2_ ball, so the composition element of the ZrO_2_ ball will be detected in the debris. In the later stage of friction, the material transfer process between two friction pair materials and the formation of debris gradually stabilize, so the friction coefficient gradually stabilizes at a higher value. Cryogenic treatment makes the Ti-6Al-4V titanium alloy more uniform in microstructure and higher in hardness, which leads to CT Ti alloy having a better wear resistance.

Under the wet condition, the liquid medium can isolate oxygen to a great extent, so the wear mechanism of titanium alloy is mainly abrasive wear and adhesive wear. Under the condition of wet wear, because of the existence of liquid medium, the temperature between friction pairs is low, and the material transfer between friction pairs is difficult, which restrains the occurrence of adhesive wear. Therefore, under the wet condition, the friction coefficient of titanium alloy samples will gradually decrease, and then the wear process will reach a stable state. The plough grooves on the NT Ti alloy surface are wider and mainly formed by the ploughing effect from the micro-convex body. These micro-convex bodies are mainly formed by the titanium alloy material transferred to the ZrO_2_ ball. The plough grooves on the surface of CT Ti alloy are fine, which is mainly induced by the micro-cutting process. The micro-convex body on the surface of the ZrO_2_ ball plays an important role in the process. In conclusion, cryogenic treatment can reduce the adhesive wear of Ti-6Al-4V titanium alloy under dry/wet friction conditions.

In this paper, the effect of cryogenic treatment on the friction and wear properties of Ti-6Al-4V titanium alloy is studied. The friction process can be regarded as a single abrasive particle scratching on the surface of titanium alloys. The force and relative velocity between the workpiece and abrasive particles are the two most important parameters in the process of barrel finishing. The main parameters of this experiment are obtained in the actual finishing process of Ti-6Al-4V titanium alloy. The experimental results show that the friction process of CT Ti alloy can rapidly stabilize under wet friction and wear conditions. It is indicated that the friction process of titanium alloy under the above conditions is more predictable and controllable. It means that through a reasonable experimental design and experimental simplification, the parameters needed for actual processing can be explored with lower costs and higher efficiency.

## 4. Conclusions

The friction and wear behavior of Ti-6Al-4V titanium alloy with and without cryogenic treatment under a low load (5 N), low scratch speed (5 cm/s) and dry/wet conditions were studied. The main conclusions are as follows:

(1) Cryogenic treatment leads to the transformation of part of the β phase into a secondary α phase in the microstructure of Ti-6Al-4V titanium alloy. The comprehensive effects of the cryogenic treatment result in improved hardness, which is helpful to enhance the friction and wear properties of the titanium alloy.

(2) After cryogenic treatment, the Ti-6Al-4V titanium alloy exhibits a lower friction coefficient and wear rate under both dry and wet wear conditions. Under wet conditions, the friction coefficient after stabilization is lower than that under dry conditions. After cryogenic treatment, the friction coefficient of Ti-6Al-4V alloy is 0.18 after reaching a stable wear stage under wet conditions. The time required for the CT Ti alloy to reach the stable wear stage is obviously shorter than that for the NT Ti alloy. Cryogenic treatment makes CT Ti alloy have a more uniform microstructure and higher microhardness than NT Ti alloy, so that the former can enter a stable friction stage in a shorter time.

(3) Under dry wear conditions, the wear mechanism of NT Ti alloy samples is typical abrasive wear, heavy adhesion wear and slight oxidation wear. The wear mechanism of CT Ti alloy samples is abrasive wear, slight oxidation wear and adhesion wear. Under wet wear conditions, the wear mechanism of NT Ti alloy samples is abrasive wear and slight adhesion wear. After cryogenic treatment, the mechanism is changed to slight abrasive wear. Cryogenic treatment improves the microhardness of Ti-6Al-4V titanium alloy and reduces its tendency of adhesion wear. With the participation of a liquid medium, the wear process of Ti-6Al-4V titanium alloy can stabilize quickly. The combination use of a cryogenic treatment and a liquid medium will make the wear process of titanium alloy more predictable and controllable.

## Figures and Tables

**Table 1:** Chemical composition of Ti-6Al-4V titanium alloy (wt. %).

Ti	Al	V	Fe	C
Bal.	6.30	4.48	0.05	0.03
